# Supplementary material for: Forest defoliator outbreaks alter nutrient cycling in northern waters
Source: Nat Commun. 2021 Nov 3;12:6355. doi: 10.1038/s41467-021-26666-1 (PMC8566564; doi:10.1038/s41467-021-26666-1)
Supplement: Supplementary file 1 — Supplementary Information [file 41467_2021_26666_MOESM1_ESM.pdf]

Supplementary Figures

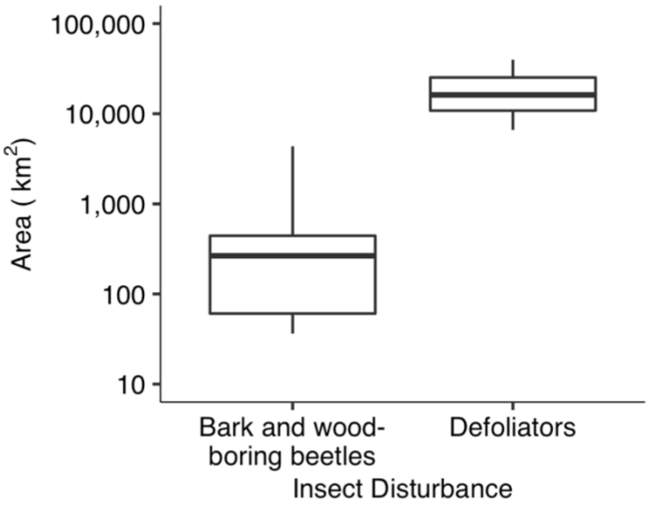

Supplementary Figure 1. Defoliating insect species annually covered 23-times the area of all bark and wood-boring beetles combined within our 439,661 km^2 study area. The area disturbed by defoliators and bark/wood-boring beetles was calculated using aerial surveys from Natural Resources Canada – Canadian Forest Service (CFS) and the Ontario Ministry of Natural Resources and Forestry (OMNRF). Solid lines show medians, boxes show inter-quartile range, and whiskers show 1.5-times the interquartile range. A paired two-sided t-test was used to compare the mean difference in the annual area covered between the two groups from 2004 to 2016 (t_{12} = - 8.00, $p < 0.001$). Data from 2004 to 2016 were used as this was when both defoliators and bark/wood-boring beetle disturbances were present in the region.

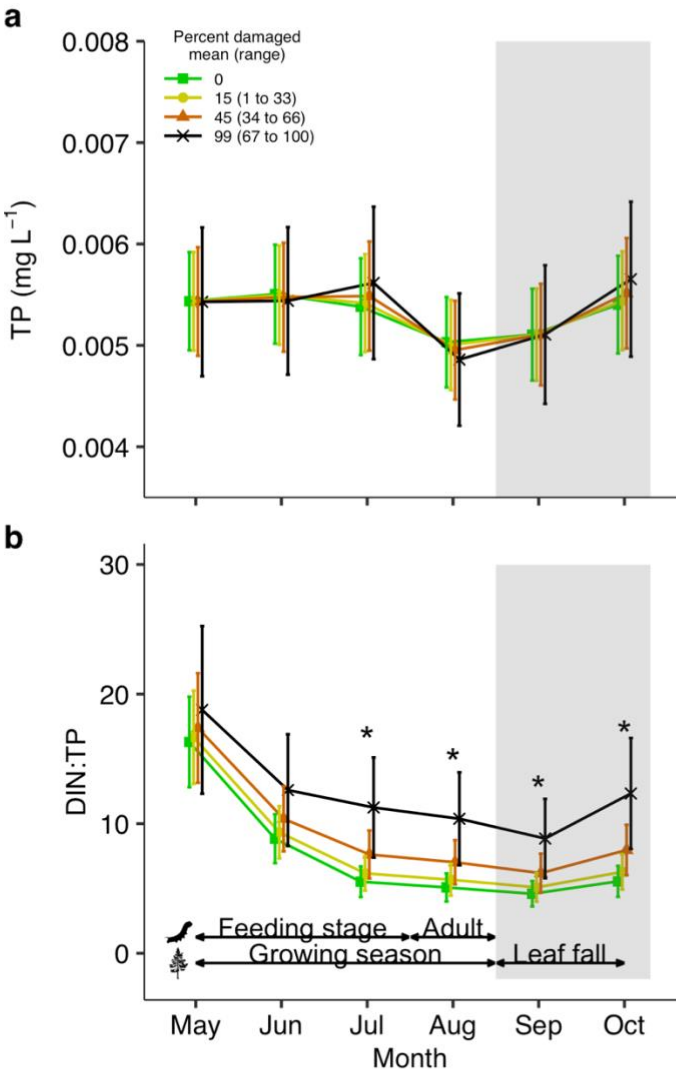

Supplementary Figure 2. Total phosphorus (TP) changed little during insect outbreaks. Mean (± 95% CI) monthly (a) TP and (b) DIN:TP ratio in lake water averaged across 12 lakes from 1985 to 2016. For visualization purposes, monthly catchment-level averages of TP and DIN:TP were grouped into three equal-width bins for the percent catchment area defoliated and points show corresponding mean ±SE. However, slopes were estimated to models fitted to non-binned data with N = 296 to 358 and 240 to 312 per month for TP and DIN:TP, respectively (Supplementary Table 2). TP did not change with insects (mean 95% CI across months = <-0.01 to <0.01) but DIN:TP ratio increased during outbreaks in

later months (mean 95% CI across months = 0.16 to 1.27). Upper arrows in (b) are the typical life cycle stages of defoliating insects while lower arrows indicate typical tree phenology. Shaded area represents typical period of leaf senescence. Asterisks (*) denotes a statistically significant effect of the percentage of catchment damage on monthly DIN:TP within a given month calculated using estimated marginal means (see Supplementary Table 2). Conditional R_c^2 = 0.41 and 0.57 in (a) and (b), respectively.

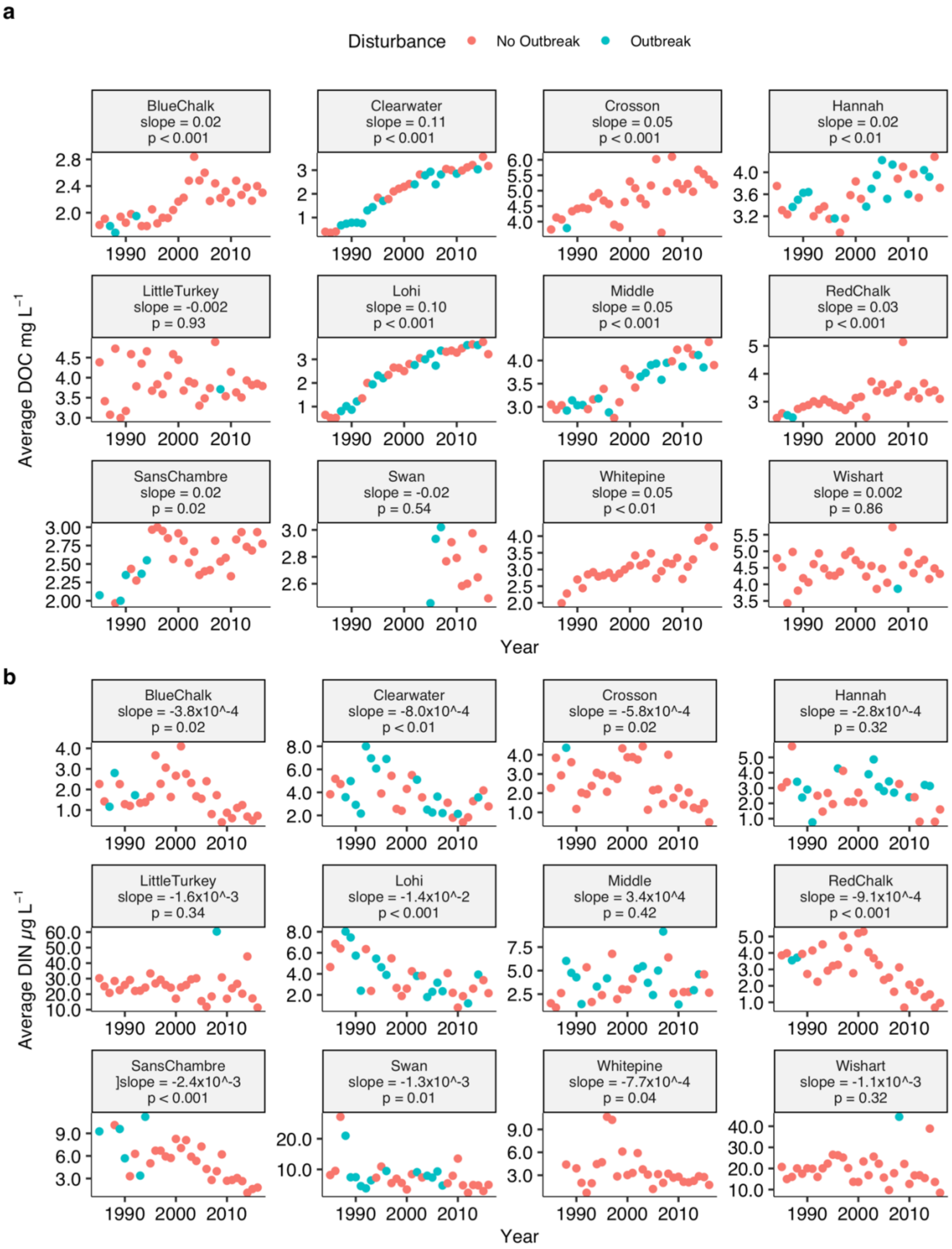
a
Disturbance
No Outbreak
Outbreak
BlueChalk
slope = 0.02
p < 0.001
Clearwater
slope = 0.11
p < 0.001
Crosson
slope = 0.05
p < 0.001
Hannah
slope = 0.02
p < 0.01
LittleTurkey
slope = -0.002
p = 0.93
Lohi
slope = 0.10
p < 0.001
Middle
slope = 0.05
p < 0.001
RedChalk
slope = 0.03
p < 0.001
SansChambre
slope = 0.02
p = 0.02
Swan
slope = -0.02
p = 0.54
Whitepine
slope = 0.05
p < 0.01
Wishart
slope = 0.002
p = 0.86
Average DOC mg L^{-1}
Year
b
BlueChalk
slope = -3.8x10^-4
p = 0.02
Clearwater
slope = -8.0x10^-4
p < 0.01
Crosson
slope = -5.8x10^-4
p = 0.02
Hannah
slope = -2.8x10^-4
p = 0.32
LittleTurkey
slope = -1.6x10^-3
p = 0.34
Lohi
slope = -1.4x10^-2
p < 0.001
Middle
slope = 3.4x10^4
p = 0.42
RedChalk
slope = -9.1x10^-4
p < 0.001
SansChambre
]slope = -2.4x10^-3
p < 0.001
Swan
slope = -1.3x10^-3
p = 0.01
Whitepine
slope = -7.7x10^-4
p = 0.04
Wishart
slope = -1.1x10^-3
p = 0.32
Average DIN μg L^{-1}
Year

Supplementary Figure 3. Temporal trends in water chemistry in the 12 study lakes from 1985 to 2016. Average ice-free (May to October) (a) DOC concentrations increased while (b) DIN concentrations decreased. Theil-Sen's slopes and p values represent the rate of change and significance in (a) DOC (mg L^{-1}) or (b) DIN (µg L^{-1}) for each catchment. Blue points indicate years with outbreaks while red represents no outbreaks.

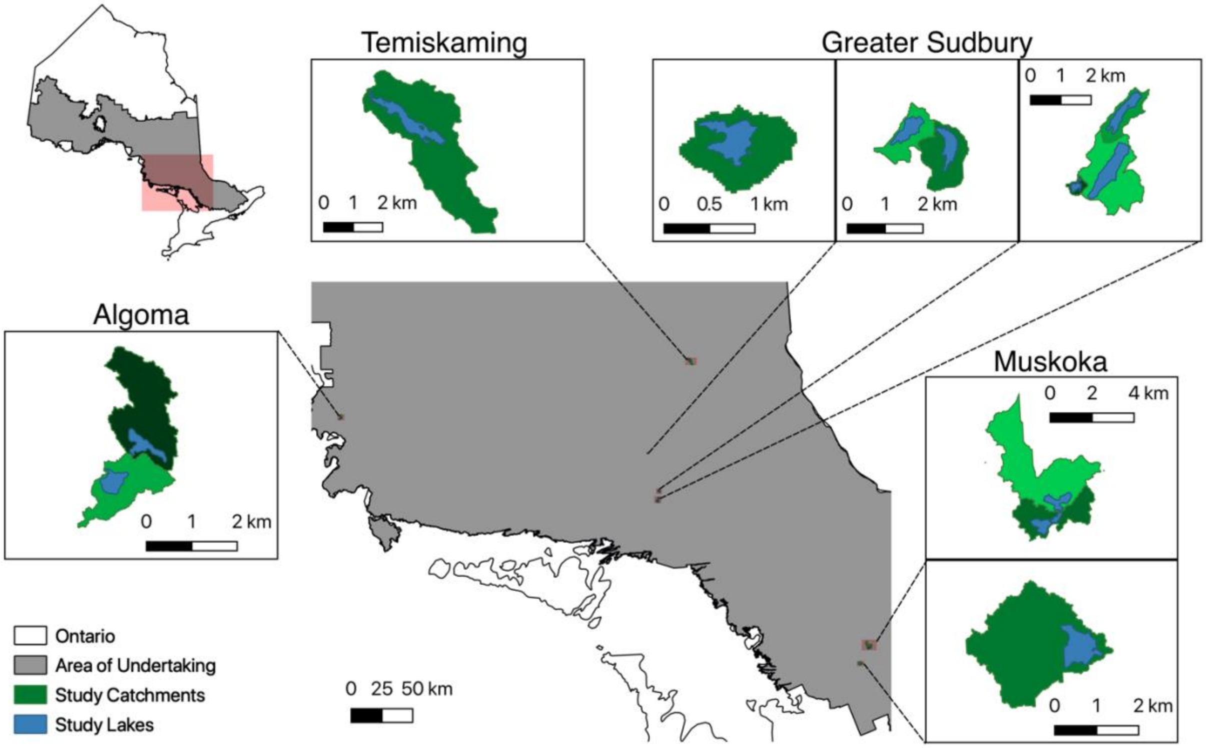

Supplementary Figure 4. Map of study catchments within our study area. The grey area represents the Area of Undertaking, where insect outbreaks were surveyed by Natural Resources Canada – Canadian Forest Service (CFS) and the Ontario Ministry of Natural Resources and Forestry (OMNRF). Catchments are denoted in green with different shades used when two or more catchments are adjoined (for visualisation).

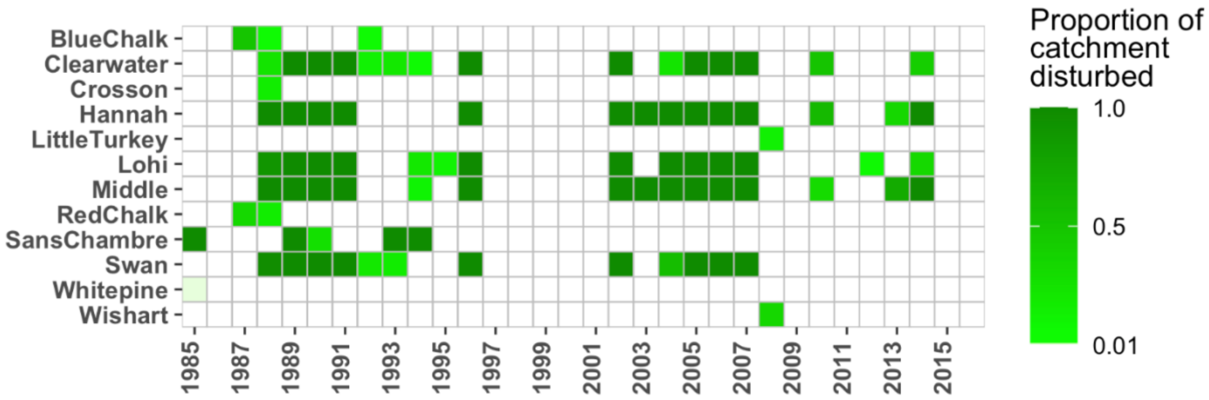

Supplementary Figure 5. History of insect outbreaks within each catchment. Green tiles represent the proportion of catchment disturbed while white tiles show years where no outbreaks occurred.

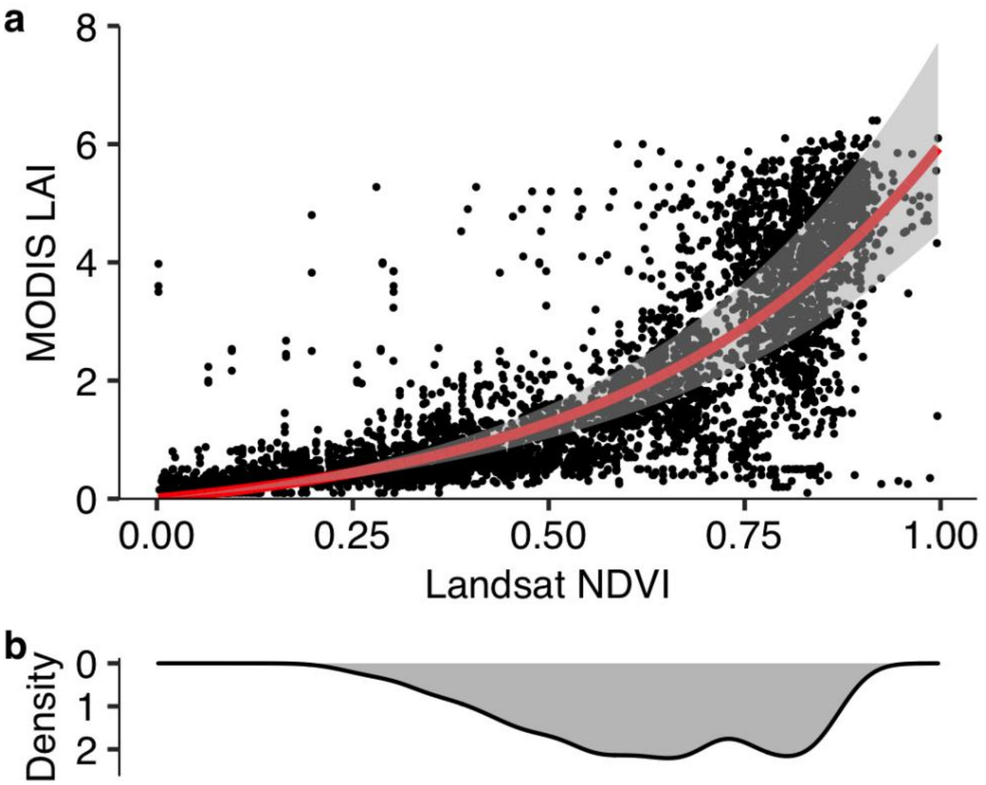

Supplementary Figure 6. Prediction of LAI from NDVI in 2016. (a) Correlation between Landsat NDVI and MODIS LAI across pixels on 23 imaging dates in our 11 study catchments (total n = 5865). 30 m^2 NDVI pixels from January to December 2016 were rescaled to match 500 m^2 LAI pixels. The red line in (a) represents the mean (±95% CI) fit between NDVI and LAI: $LAI = 0.48 \times (e^{2.6 \times NDVI} - 1)$. (b) Probability distribution of NDVI values in our catchments.

Supplementary Table 1. Overall model summary outputs for the effects of insect damage, month, and forest cover on LAI, DOC and DIN across our 12 study catchments/lakes. All continuous independent variables were scaled to a mean of zero and standard deviation of one to compare effect sizes. Model estimates and 95% confidence intervals (in parentheses) are displayed along with the associated p-values. Significant p-values indicate a statistical difference from the model intercept.

	**LAI**		**DOC**		**log(DIN)**	
Predictors	*Estimates*	*p*	*Estimates*	*p*	*Estimates*	*p*
Intercept	1.69<br>(1.54 – 1.84)	**<0.001**	2.92<br>(2.44 – 3.40)	**<0.001**	-2.42<br>(-2.86 – -1.98)	**<0.001**
Percent Disturbed	-0.06<br>(-0.16 – 0.03)	0.208	-0.17<br>(-0.35 – 0.02)	0.074	0.06<br>(-0.12 – 0.24)	0.501
Month [Jun]	1.15<br>(1.11 – 1.19)	**<0.001**	0.18<br>(0.11 – 0.25)	**<0.001**	-0.56<br>(-0.64 – -0.47)	**<0.001**
Month [Jul]	1.30<br>(1.25 – 1.35)	**<0.001**	0.31<br>(0.22 – 0.40)	**<0.001**	-0.97<br>(-1.07 – -0.87)	**<0.001**
Month [Aug]	1.07<br>(1.01 – 1.13)	**<0.001**	0.27<br>(0.17 – 0.37)	**<0.001**	-1.15<br>(-1.26 – -1.05)	**<0.001**
Month [Sep]	0.03<br>(-0.03 – 0.09)	0.349	0.30<br>(0.19 – 0.40)	**<0.001**	-1.23<br>(-1.34 – -1.12)	**<0.001**
Month [Oct]	-0.69<br>(-0.75 – -0.63)	**<0.001**	0.29<br>(0.18 – 0.40)	**<0.001**	-0.96<br>(-1.07 – -0.84)	**<0.001**

Proportion deciduous trees	0.36<br>(0.19 – 0.54)	**0.001**	0.32<br>(-0.26 – 0.90)	0.246	0.42<br>(-0.11 – 0.95)	0.108
Proportion coniferous trees	0.21<br>(0.04 – 0.39)	**0.022**	0.07<br>(-0.50 – 0.64)	0.791	-0.03<br>(-0.47 – 0.42)	0.901
Percent disturbed × Month [Jun]	-0.19<br>(-0.23 – -0.15)	**<0.001**	0.02<br>(-0.05 – 0.09)	0.613	0.06<br>(-0.02 – 0.15)	0.134
Percent disturbed × Month [Jul]	-0.20<br>(-0.25 – -0.14)	**<0.001**	-0.08<br>(-0.17 – 0.01)	0.075	0.20<br>(0.10 – 0.30)	**<0.001**
Percent disturbed × Month [Aug]	-0.12<br>(-0.18 – -0.06)	**<0.001**	-0.05<br>(-0.15 – 0.05)	0.304	0.20<br>(0.09 – 0.31)	**<0.001**
Percent disturbed × Month [Sep]	0.05<br>(-0.01 – 0.11)	0.120	-0.11<br>(-0.21 – -0.01)	**0.038**	0.18<br>(0.07 – 0.29)	**0.001**
Percent disturbed × Month [Oct]	0.11<br>(0.04 – 0.17)	**0.001**	-0.09<br>(-0.20 – 0.02)	0.117	0.24<br>(0.12 – 0.35)	**<0.001**
Percent disturbed × Proportion deciduous	-0.02<br>(-0.13 – 0.09)	0.740	-0.24<br>(-0.45 – -0.03)	**0.028**	0.09<br>(-0.09 – 0.27)	0.306
Percent disturbed × Proportion coniferous	-0.04<br>(-0.14 – 0.06)	0.460	0.05<br>(-0.20 – 0.30)	0.717	0.02<br>(-0.13 – 0.16)	0.832
Observations	2154		1803		1659	

Supplementary Table 2. Within-month effects of percentage of catchment damaged on LAI, DOC, DIN, TP, and DIN:TP. Values were computed using the *emmeans* package in R. Modelled estimates and 95% confidence intervals (in parentheses) are displayed along with associated p-values. Bolded p-values indicate statistically significant effects, i.e., different from zero.

	**LAI**		**DOC (mg L^{-1})**		**DIN (mg L^{-1})**		**TP (mg L^{-1})**		**DIN:TP**	
Month	*Estimates*	*p*	*Estimates*	*p*	*Estimates*	*p*	*Estimates*	*p*	*Estimates*	*p*
May	-0.18 (-0.46 – 0.10)	0.208	-0.48 (-1.00 – 0.04)	0.071	0.17 (-0.33 – 0.67)	0.501	-0.001 (-0.22 - 0.21)	0.992	0.14 (-0.42 - 0.69)	0.623
June	-0. 71 (-0.99 – -0.43)	**<0.001**	-0.44 (-0.95 – 0.08)	0.096	0.35 (-0.15 – 0.85)	0.166	-0.01 (-0.22 - 0.20)	0.909	0.34 (-0.21 - 0.89)	0.222
July	-0. 74 (-1.02 – -0.46)	**<0.001**	-0.72 (-1.24 – -0.21)	**0.006**	0.73 (0.24 – 1.23)	**0.004**	0.04 (-0.17 - 0.25)	0.687	0.72 (0.17 - 1.27)	**0.010**
Aug	-0. 52 (-0.80 – -0.24)	**<0.001**	-0.64 (-1.16 – -0.12)	**0.015**	0.73 (0.23 –1.24)	**0.004**	-0.03 (-0.25 - 0.40)	0.745	0.71 (0.16 - 1.27)	**0.012**
Sep	-0.05 (-0.31 – 0.24)	0.753	-0.85 (-1.37 – -0.33)	**0.001**	0.68 (0.18 – 1.19)	**0.008**	0.0002 (-0.21 - 021)	0.998	0.65 (0.09 - 1.20)	**0.023**
Oct	0.12 (-0.16 – 0.40)	0.390	-0.74 (-1.26 – -0.22)	**0.005**	0.85 (0.34 – 1.36)	**0.001**	0.05 (-0.17 - 0.26)	0.673	0.77 (0.21 - 1.33)	**0.008**

Supplementary Table 3. Summary of study lakes. Asterisk (*) denotes lake that is not thermally stratified.

**Region**	**Lake**	**Latitude (°)**	**Longitude (°)**	**Lake Area (ha)**	**Max depth (m)**	**Catchment Area (ha)**
Algoma	Little Turkey	47.04227	-84.40812	19.85	13.0	134
Algoma	Wishart*	47.04948	-84.39924	19.09	4.5	245
Greater Sudbury	Clearwater	46.37042	-81.05045	77.02	21.5	285
Greater Sudbury	Hannah	46.44328	-81.03831	27.80	8.0	83
Greater Sudbury	Lohi	46.38749	-81.04330	41.15	19.0	83
Greater Sudbury	Middle	46.43897	-81.02482	28.98	12.0	132
Greater Sudbury	Sans Chambre	46.72141	-81.13066	15.72	17.5	62
Greater Sudbury	Swan	46.36602	-81.06544	7.48	8.8	18
Muskoka	Blue Chalk	45.19917	-78.93835	49.55	22.0	1045
Muskoka	Crosson	45.08356	-79.03645	54.96	26.0	508
Muskoka	Red Chalk	45.18981	-78.94753	55.10	32.0	381
Temiskaming	Whitepine	47.38400	-80.63133	87.24	59.0	911

Supplementary Table 4. Summary of defoliating insect surveyed by CFS and OMNRF. Bolded species were present in our 12 lake catchments. Outbreaks were defined as ≥50% of the catchment affected.

Common Name	Species Name	Years With Recorded Outbreaks	Unique Catchments Affected
**Forest Tent Caterpillar**	*Malacosoma disstria*	1948-57, 1961-2018	55097
**Spruce Budworm**	*Choristoneura fumiferana*	1941-63, 1967-2018	53667
**Jack Pine Budworm**	*Choristoneura pinus*	1937-1942, 1945-48, 1954, 1961-62, 1967-73, 1983-97, 2003-12, 2015-18	22391
Birch Skeletonizer	*Bucculatrix canadensisella*	2002, 2005, 2012	14677
**Aspen Twoleaf Tier**	*Enargia decolor*	1997-96, 2005-06, 2010-14	8370
Large Aspen Tortrix	*Choristoneura conflictana*	1996-2002, 2004-12, 2016-18	3472
**Gypsy Moth**	*Lymantria dispar dispar*	1981-96, 1999-2004, 2006, 2008, 2012-14	2675
**Bruce Spanworm**	*Operophtera bruceata*	1998, 2000, 2002-03, 2008-10	850
Poplar Serpentine Leafminer	*Phyllocnistis populiella*	1997	318
Larch Casebearer	*Coleophora laricella*	2002-07, 2009-17	238
Cedar Leafminer	*Argyresthia canadensis*	2003, 2005, 2007, 2010-12, 2014-15, 2017-18	228
Unknown Aspen Leafroller	NA	2007, 2010	185
Early Aspen Leafroller	*Pseudexentera oregonana*	2008, 2011	162
Maple Leafcutter	*Paraclemensia acerifoliella*	2004-05	109
Hemlock Looper	*Lambdina fiscellaria*	2001-2005	100
Oak Defoliators Complex	*Croesia semipurpurana*, *Choristoneura rosaceana*	2007, 2009	75
Poplar Flea Beetle	*Altica populi*	2002, 2005	61
Introduced Pine Sawfly	*Diprion similis*	2000	37
Redhumped Oakworm	*Symmerista canicosta*	2004-05	22
Birch Leafminer	*Fenusa pusilla*	2002, 2013	12
Other Insect	NA	2013, 2016, 2018	9
Birch Casebearer	*Coleophora serratella*	2012	4
Oak Leaf Roller	*Argyrotaenia quercifoliana*	2006	4

Pine False Webworm	*Acantholyda erythrocephala*	2009-13, 2015, 2017	4
Pink-striped Oakworm	*Anisota virginiensis*	2009-10, 2012	4
Fall Cankerworm	*Alsophila pometaria*	2002	2
Fall Webworm	*Hyphantria cunea*	2016	1
Satin Moth	*Leucoma salicis*	2018	1
